# Sialic acid serves as a functional receptor for grass carp reovirus

**DOI:** 10.1371/journal.ppat.1013481

**Published:** 2025-09-05

**Authors:** Qian Wang, Zichao Peng, Bin Gui, Yongming Li, Lanjie Liao, Zuoyan Zhu, Yaping Wang, Libo He

**Affiliations:** 1 State Key Laboratory of Breeding Biotechnology and Sustainable Aquaculture, Institute of Hydrobiology, Chinese Academy of Sciences, Wuhan, China; 2 University of Chinese Academy of Sciences, Beijing, China; Vanderbilt University Medical Center, UNITED STATES OF AMERICA

## Abstract

Grass carp reovirus (GCRV) causes hemorrhagic disease and substantial economic losses in the aquaculture of grass carp (*Ctenopharyngodon idella*), a commercially important fish species in China. Although viral entry depends on interactions between viral proteins and host receptors, the specific host molecules mediating this process have not been fully elucidated. Here, we identify cell surface sialic acid (SA) as a critical functional receptor for GCRV. Enzymatic removal of SA markedly impaired viral attachment and infection. Competitive inhibition using SA-binding lectins or soluble SA confirmed that GCRV targets SA moieties on host cells. Genetic knockdown of SA biosynthesis attenuated viral binding and replication, whereas overexpression of SA pathway genes enhanced susceptibility. Surface plasmon resonance demonstrated direct binding between GCRV capsid proteins and soluble SA, and mutational analysis identified key amino acid residues involved. Notably, pretreatment of GCRV with soluble SA significantly improved fish survival and reduced virus-induced immune overactivation *in vivo*. To assess receptor specificity, parallel experiments using *Rana grylio* virus (RGV), a phylogenetically unrelated *ranavirus*, showed that RGV infection was unaffected by SA-targeted interventions, highlighting the specificity of SA utilization by GCRV. Together, these findings identify SA as a functional and specific receptor for GCRV, offering new insights into virus-host interactions and potential antiviral strategies in aquaculture.

## Author summary

Grass carp hemorrhagic disease, caused by grass carp reovirus (GCRV), poses a major threat to freshwater aquaculture in China. Despite extensive studies on GCRV, the identity of host cell receptors mediating viral entry remained unknown. In this study, we demonstrate that GCRV directly binds to sialic acids-sugar molecules commonly found on cell surfaces-to attach to and infect host cells. By using a combination of enzymatic removal, lectin blocking, genetic manipulation, and biochemical binding assays, we show that sialic acid is essential for GCRV attachment and infection. Furthermore, pre-incubating GCRV with soluble sialic acids significantly reduced infection in cultured cells and improved the survival of infected grass carp. These findings reveal a key mechanism of viral entry and suggest new strategies for disease prevention and selective breeding of resistant fish. Our study not only deepens understanding of aquareovirus biology but also provides practical insights for controlling viral outbreaks in aquaculture.

## Introduction

Grass carp hemorrhagic disease, caused by grass carp reovirus (GCRV), represents a major threat to the aquaculture industry in China. As the most virulent member of the *Aquareovirus* genus (*Spinareoviridae* family), GCRV has been classified into three genotypes: type I (e.g., GCRV-873), type II (e.g., GCRV-HZ08), and type III (e.g., GCRV-104) [1–3]. GCRV-873 (type I) was the first fish virus sequenced in China and has served as a model strain for early molecular studies [4]. However, recent epidemiological data indicate that type II strains, including GCRV-HZ08, GCRV-GD108, and GCRV-109, are currently predominant in southern China [3,5,6]. In contrast, type III appears geographically restricted, with only GCRV-104 reported to date [7].

Despite sharing an 11-segment double-stranded RNA genome, the three GCRV genotypes exhibit considerable differences in virion architecture, protein composition, and pathogenicity. GCRV-I encodes seven structural and five nonstructural proteins, with VP5 and VP7 as the outer capsid proteins mediating viral entry [8–11]. GCRV-II encodes nine structural and two nonstructural proteins, with VP4, VP56, and VP35 predicted to function as outer capsid proteins [6,12]. GCRV-III encodes 12 viral proteins, with only VP4 identified as the outer capsid protein [7]. Sequence divergence among genotypes is substantial, with less than 20% amino acid sequence identity across many gene segments [3], suggesting potential differences in cell entry mechanisms and host interactions.

Viral entry is initiated by interactions between outer capsid/envelope proteins and specific cell surface receptors [13,14]. These receptor interactions determine viral tropism, infectivity, and host specificity, and are therefore critical targets for disease prevention and control [15,16]. Given the significant economic losses caused by GCRV, considerable efforts have been devoted to elucidating its pathogenesis and to developing disease-resistant breeding strategies [17–21]. Several host proteins have been reported to interact with GCRV-encoded proteins and modulate infection [11,22–27], yet whether these proteins serve as primary cellular receptors remains unknown. Thus, the key receptors mediating GCRV infection have yet to be clearly defined.

In this study, we identified cell surface sialic acid (SA) as a functional receptor for GCRV. Enzymatic removal of SA significantly reduced viral binding and infection. SA-binding lectins and soluble SA competitively inhibited GCRV attachment, while genetic manipulation of SA biosynthesis modulated viral susceptibility. Surface plasmon resonance assays confirmed direct binding between GCRV capsid proteins and SA, and *in vivo* neutralization assays demonstrated that soluble SA pretreatment increased survival and reduced immune overactivation in GCRV infected grass carp. These findings reveal the molecular basis of GCRV-host cell interactions and suggest SA as a promising antiviral target in aquaculture.

## Results

### Enzymatic removal of SA markedly diminished viral attachment and subsequent infection

Sialic acid (SA) is a well-characterized attachment and entry receptor for numerous viruses [28–30]. To investigate whether GCRV utilizes SA as a functional receptor, we first examined the distribution of SAs on the surfaces of two grass carp cell lines, grass carp ovary (GCO) and *Ctenopharyngodon idella* kidney (CIK) cells, using immunofluorescence (IF) staining. FITC-conjugated WGA (to stain total SA), MALII (to stain α2,3-linked SA), and SNA (to stain α2,6-linked SA) were employed. As shown in S1 Fig, both cell types exhibited abundant distribution of total SA, α2,3-linked SA, and α2,6-linked SA (S1A and S1B Fig), with nearly all cells stained by the three lectins, confirming the widespread presence of SA in both grass carp cell lines.

**Fig 1:**
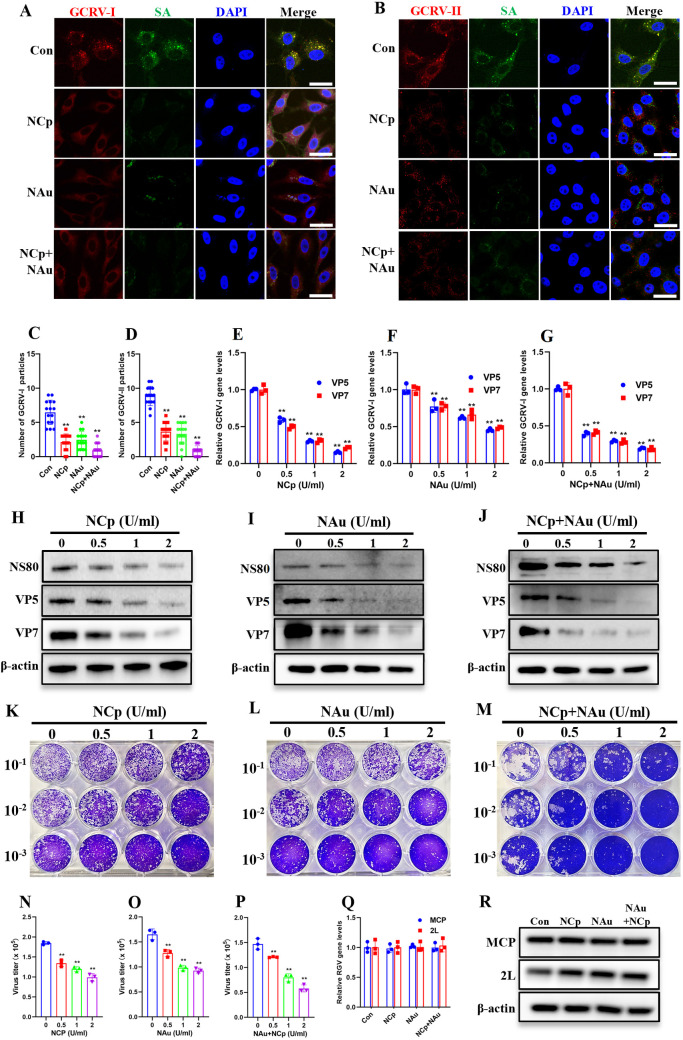
Enzymatic removal of SA markedly diminished viral attachment and subsequent infection. **(A, B)** Immunofluorescence analysis of neuraminidase-treated cells incubated GCRV. Cells were either untreated or treated with neuraminidases (2 U/ml, 2 h, 28 °C), followed by incubation with GCRV-I (A) or GCRV-II (B) (MOI = 100, 1h, 4 °C). Cells were then stained with FITC-conjugated WGA and antibodies against GCRV outer capsid proteins (VP5 for GCRV-I; VP35 for GCRV-II). Scale bar = 10 µm. **(C, D)** Quantification of attached GCRV-I (C) or GCRV-II (D) particles by ImageJ software. **(E-G)** RT-qPCR analysis of relative GCRV-I gene levels in neuraminidase-treated or untreated cells incubated with GCRV-I. **(H-J)** Western blotting analysis of NS80, VP5, and VP7 in neuraminidase-treated or untreated cells infected with GCRV-I. **(K-M)** Plaque assay of neuraminidase-treated or untreated cells infected with GCRV-I. **(N-P)** Calculated virus titers in neuraminidase-treated or untreated cells. **(Q)** RT-qPCR analysis of the relative RGV gene levels in neuraminidase-treated or untreated cells incubated with RGV. **(R)** Western blotting analysis of MCP and 2L in neuraminidase-treated or untreated cells infected with RGV. NCp: neuraminidase from *C. perfringens*, NAu: neuraminidase from *A. ureafaciens*. Data are represented as mean (n = 15 for C and D, n = 3 for E-G and N-Q) ± SD. ** indicates *P* < 0.01.

We investigated SA’s function in GCRV attachment by enzymatically stripping surface SAs from CIK cells using neuraminidases treatment. Neuraminidase from *C. perfringens* (preferentially removing α2,3-linked SA), or from *A. ureafaciens* (preferentially removing α2,6-linked SA), or a combination of both were applied at varying concentrations at 28°C for 2 hours. IF staining with FITC-conjugated WGA confirmed the efficient removal of SAs by both neuraminidases, with a slight additive effect observed when both were combined (S2A-S2C Fig). Flow cytometry (FCM) yielded similar results (S2D-S2F Fig). We detected no cytotoxicity in cells treated with all neuraminidases for 2 hours at 28°C at any concentration tested (S2G-S2I Fig). Given the limited spread of type III GCRV (7), we focused on GCRV-I and GCRV-II in this study. Accordingly, GCRV-I and GCRV-II were incubated with either neuraminidase-treated or untreated cells at 4°C for 1h to permit viral attachment while preventing subsequent infection. Then, cells were dual-stained with FITC-WGA and GCRV-specific antibodies (anti-VP5 for GCRV-I; anti-VP35 for GCRV-II) for IF analysis. Results revealed neuraminidases pretreatment significantly diminished GCRV attachment, as evidenced by reduced fluorescent particle counts relative to untreated cells (Fig 1A and 1B). Interestingly, high-resolution imaging (S2J Fig) and quantitative colocalization analysis (S2K and S2L Fig) demonstrated strong spatial overlap between SA (green fluorescence) and GCRV virions (red fluorescence), with Pearson colocalization coefficients (PCC) of 0.7320 for GCRV-I and 0.7148 for GCRV-II, respectively. This suggests that these GCRV virions are attached to the cell surface. Furthermore, three-dimensional (3D) Z-section reconstruction analysis of the stained cells confirmed the viral particles are indeed located on the cell surface (S1 Movie). To quantify GCRV attachment following neuraminidases treatment, we enumerated antibody-labeled viral particles on cell surfaces across 15 cells per group using ImageJ software. Neuraminidases treatment significantly decreased GCRV-I attachment from 6.47 particles/cell (untreated) to 2.13, 2.47, and 0.93 particles/cell in treated groups (Fig 1C). Similar results were observed for GCRV-II (Fig 1D). qPCR analysis further confirmed that neuraminidases treatment significantly reduced GCRV attachment in a dose-dependent manner (Figs 1E-1G and S2M-S2O).

Since virus attachment is the initial step in infection, its reduction should also affect the subsequent infection process. Given that GCRV-II infection fails to produce observable CPE in CIK cells [31], we specifically examined the impact of neuraminidases treatment on GCRV-I infection. Following infection with GCRV-I (MOI = 1) for 24 h, neuraminidase-treated and untreated cells were harvested for analysis. GCRV-I protein levels (NS80, VP5, VP7) showed dose-dependent reduction following neuraminidases treatment (Fig 1H-1J). Plaque assays using supernatants from treated or untreated cells further demonstrated a significant reduction in GCRV-I plaque numbers (Fig 1K-1M) and virus titers (Fig 1N-1P) following neuraminidases treatment.

To validate SA’s specific role in GCRV infection, we performed parallel experiments using *Rana grylio* virus (RGV), a *ranavirus* (*Iridoviridae* family) that causes lethal infections in pig frogs but is phylogenetically distinct from GCRV [32,33]. There is no evidence to suggest that SA functions as a functional receptor for RGV. RGV infection was performed at 4°C for 1 h (for qPCR analysis) or 28°C for 24 h (for western blotting) using both neuraminidase-treated and untreated cells. In contrast to GCRV, RGV showed no dependence on SA for viral attachment or infection, as neuraminidases treatment produced no significant change in viral binding or protein expression (Fig 1Q and 1R), indicating that the effect of neuraminidases treatment is specific to GCRV. Collectively, these results indicate that enzymatic removal of SA markedly diminished viral attachment and subsequent infection.

### Sialic acid-binding lectins inhibit GCRV binding to cells

**Fig 2:**
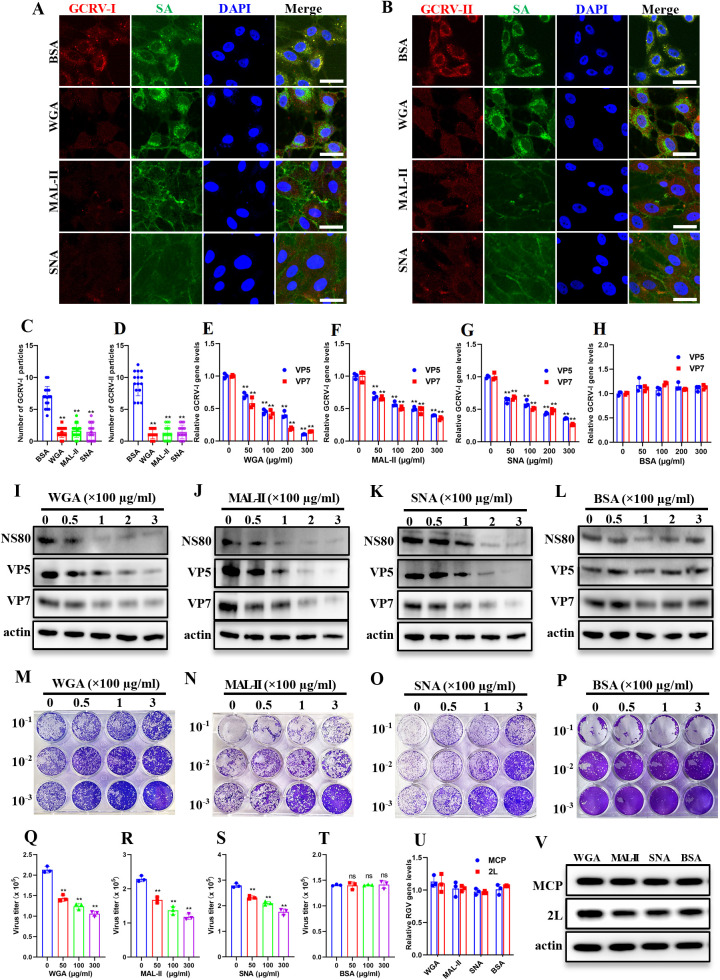
Sialic acid-binding lectins inhibit GCRV binding to cells. **(A, B)** Immunofluorescence analysis of lectin- or BSA-treated cells incubated GCRV. Cells were treated with three SA-binding lectins or BSA (300 μg/ml, 2h, 28°C), followed by incubation with GCRV-I (A) or GCRV-II (B) (MOI = 100, 1h, 4 °C). Cells were stained with FITC-conjugated WGA and antibodies against GCRV outer capsid proteins (VP5 for GCRV-I; VP35 for GCRV-II). Scale bar = 10 µm. **(C, D)** Quantification of attached GCRV-I (C) or GCRV-II (D) particles using ImageJ software. **(E-H)** RT-qPCR analysis of the relative GCRV-I gene levels in lectin- or BSA-treated cells incubated with GCRV-I. **(I-L)** Western blotting analysis of NS80, VP5, and VP7 in lectin- or BSA-treated cells infected with GCRV-I. **(M-P)** Plaque assay of lectin- or BSA-treated cells infected with GCRV-I. **(Q-T)** Calculated virus titers in lectin- or BSA-treated cells. **(U)** RT-qPCR analysis of the relative RGV gene levels in lectin- or BSA-treated cells incubated with RGV. **(V)** Western blotting analysis of MCP and 2L in lectin- or BSA-treated cells infected with RGV. Data are represented as mean (n = 15 for C and D, n = 3 for E-H and Q-U) ± SD. ** indicates *P* < 0.01, ns indicates no signiﬁcant difference.

To further characterize SA’s involvement in GCRV infection, CIK cells were pretreated with three lectins targeting distinct SA configurations: WGA (total SA), MAL-II (α2,3-SA specific), and SNA (α2,6-SA specific). These lectins competitively bind to SAs on the cell surface. Cells treated with BSA served as a negative control. Lectin-treated cells were exposed to GCRV at 4°C for 1 h to permit viral attachment while blocking membrane fusion and internalization. After incubation, the cells were washed and analyzed via IF. As shown in Fig 2, GCRV particles, represented by fluorescent spots stained with antibodies on the cell surface, were almost entirely absent in three lectin-treated cells. This result indicates both GCRV-I and GCRV-II attachment were markedly impaired by SA-binding lectins (Fig 2A and 2B). Consistent with fluorescence imaging, quantitative analysis demonstrated all three lectins significantly diminished cell-associated GCRV particles (Fig 2C and 2D). qPCR quantification revealed dose-dependent inhibition of GCRV attachment by all lectins, while BSA controls showed no significant effect (Figs 2E-2H and S3A-S3D). We detected no cytotoxicity in cells treated with three lectins or BSA for 2 hours at 28°C at any concentration tested (S3E-S3H Fig).

To assess the impact of SA-binding lectins on GCRV-I infection, the lectin- or BSA-treated cells were infected with GCRV-I for 24 hours and then harvested for analyzed. Western blotting demonstrated dose-dependent reductions in GCRV-I protein expression (NS80, VP5, and VP7) following lectins treatment (Fig 2I-2L). Similarly, plaque assays demonstrated a significant, dose-dependent reduction in plaque numbers (Fig 2M-2P) formed by the supernatants of lectin-treated cells, as well as the calculated virus titers (Fig 2Q-2T), whereas BSA-treated cells showed no reduction. Moreover, the lectin- or BSA-treated cells were infected with RGV at 4°C for 1 hour or at 28°C for 24 hours, followed by qPCR or western blotting analysis. Results showed that treatment with three lectins had no effect on RGV attachment or subsequent infection (Fig 2U and 2V). Taken together, these results confirm that SA-binding lectins effectively inhibit GCRV binding to cells.

### Pretreating GCRV with soluble SAs impairs its attachment and infection

**Fig 3:**
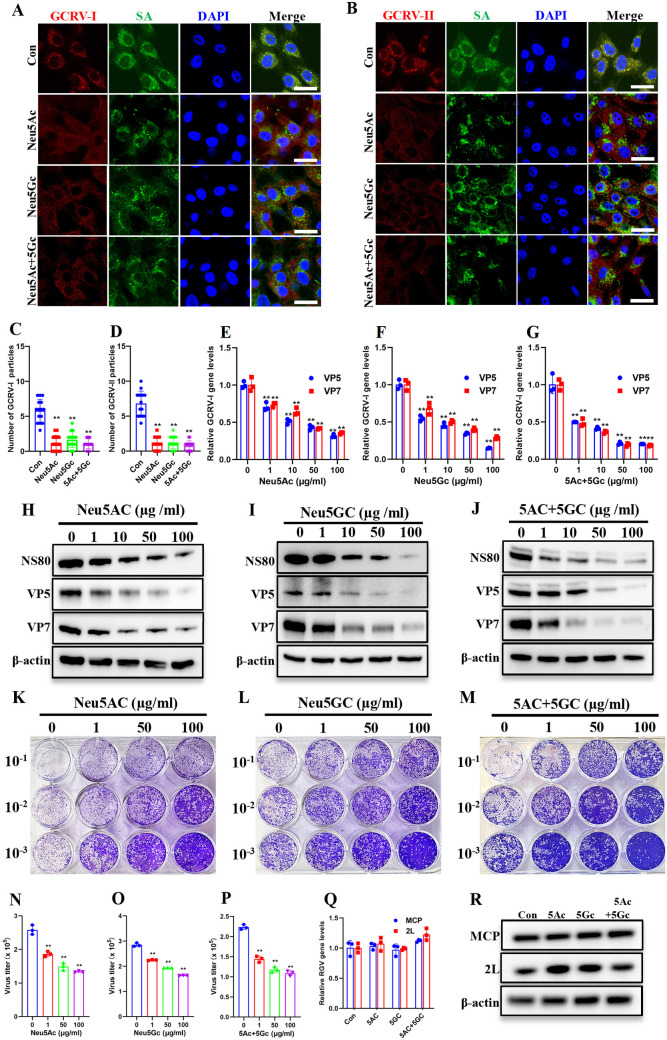
Pretreating GCRV with soluble SAs impairs its attachment and infection. **(A, B)** Immunofluorescence analysis of cells incubated with soluble SA- pretreated GCRV. CIK cells were incubated with soluble SA (100 μg/mL)-pretreated GCRV-I (A) or GCRV-II (B) (MOI = 100, 1h, 4 °C), and then cells were stained with FITC-conjugated WGA and antibodies against GCRV outer capsid proteins (VP5 for GCRV-I; VP35 for GCRV-II). Scale bar = 10 µm. **(C, D)** Quantitative analysis of attached GCRV-I (C) or GCRV-II (D) particles using ImageJ software. **(E-G)** RT-qPCR analysis of the relative GCRV-I gene levels in cells incubated with soluble SA-pretreated GCRV-I. **(H-J)** Western blotting analysis of NS80, VP5, and VP7 in cells infected with soluble SA-pretreated GCRV-I. **(K-M)** Plaque assay of cells infected with soluble SA-pretreated GCRV-I. **(N-P)** Calculated virus titers in cells infected with soluble SA-pretreated GCRV-I. **(Q)** RT-qPCR analysis of the relative RGV gene levels in cells incubated with soluble SA-pretreated or untreated RGV. **(R)** Western blotting analysis of MCP and 2L in cells infected with soluble SA-pretreated or untreated RGV. Data are represented as mean (n = 15 for C and D, n = 3 for E-G and N-Q) ± SD. ** indicates *P* < 0.01.

GCRV suspensions were preincubated with two soluble SAs, N-acetylneuraminic acid (Neu5Ac) and N-glycolylneuraminic acid (Neu5Gc), at varying concentrations at 4°C for overnight. The virus-SA mixtures were applied to CIK cells and incubated at 4°C for 1 h to permit receptor binding while preventing membrane fusion and viral entry. The samples were then analyzed via IF and qPCR. As shown in Fig 3, IF revealed that GCRV particles, represented by fluorescent spots stained with antibodies on the cell surface, were scarcely observed in the Neu5Ac, Neu5Gc, and combined Neu5Ac + Neu5Gc preincubated groups (Fig 3A and 3B). Quantification revealed soluble SAs pretreatment significantly decreased cell-associated GCRV particles when compared to untreated controls (Fig 3C and 3D). Consistently, qPCR quantification demonstrated dose-dependent inhibition of viral attachment by soluble SAs (Figs 3E-3G and S3I-S3K). We detected no cytotoxicity in cells infected with virus preincubated with soluble SAs, compared with virus without preincubation, at any concentration tested (S3L-S3N Fig).

To assess the impact on subsequent infection processes, we analyzed the effect of soluble SAs pretreatment on GCRV-I infection. Western blotting demonstrated dose-dependent reductions in GCRV-I protein expression (NS80, VP5, and VP7) following soluble SAs pretreatment (Fig 3H-3J). Similarly, plaque assays showed that the number of plaques (Fig 3K-3M) formed by the supernatants from soluble SAs-pretreated groups, as well as the calculated virus titers (Fig 3N-3P), were significantly reduced in a dose-dependent manner. Moreover, RGV was also preincubated with two soluble SAs. Cells were then infected with the preincubated mixtures at 4°C for 1 h or at 28°C for 24 h, followed by qPCR or western blotting analysis. The results showed that pretreatment with soluble SAs had no effect on RGV attachment or subsequent infection (Fig 3Q and 3R). In summary, these findings confirm that pretreating GCRV with soluble SAs effectively impairs its ability to attach to cells and initiate infection.

### Genetic knockdown of SA biosynthesis significantly impaired GCRV binding capacity and infectivity

**Fig 4:**
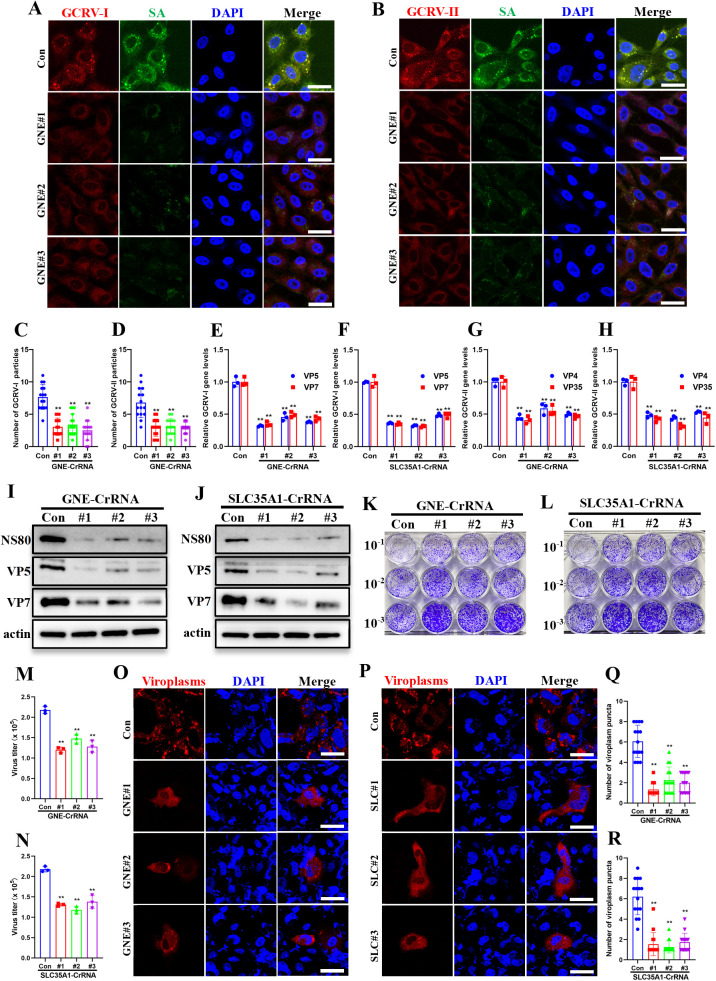
Genetic knockdown of SA biosynthesis significantly impaired GCRV binding capacity and infectivity. **(A, B)** Immunofluorescence analysis of control or GNE-knockdown cells incubated with GCRV. The control or GNE-knockdown CIK cells were incubated with GCRV-I (A) or GCRV-II (B) (MOI = 100, 1h, 4 °C), then cells were stained with FITC-conjugated WGA and antibodies against GCRV outer capsid proteins (VP5 for GCRV-I; VP35 for GCRV-II). Scale bar = 10 µm. **(C, D)** Quantification of attached GCRV-I (C) or GCRV-II (D) particles using ImageJ software. **(E-H)** RT-qPCR analysis of the relative GCRV-I gene levels in control or GNE- or SLC35A1-knockdown cells incubated with GCRV-I (E, F) or GCRV-II (G, H). **(I, J)** Western blotting analysis of NS80, VP5, and VP7 in control or GNE- (I) or SLC35A1-knockdown (J) cells infected GCRV-I. **(K, L)** Plaque assay of control or GNE- (K) or SLC35A1-knockdown (L) cells infected with GCRV-I. **(M, N)** Calculated virus titers in control or GNE- (M) or SLC35A1-knockdown (N) cells. **(O, P)** Immunofluorescence analysis of the viroplasms in control or GNE- (O) or SLC35A1-knockdown (P) cells infected with GCRV-I. Cells were infected with GCRV-I (MOI = 1, 24h, 28°C) and viroplasms were stained with antibody against NS80. Scale bar = 10 µm. **(Q, R)** Quantitative analysis of viroplasm puncta in control or GNE- (Q) or SLC35A1-knockdown (R) cells. Data are represented as mean (n = 15 for C, D, Q, and R, n = 3 for E-H, M, and N) ± SD. ** indicates *P* < 0.01.

To systematically examine SA’s role in GCRV infection, we utilized a CRISPR-Cas13d system [34] to knock down GNE and SLC35A1, two key genes in SA biosynthesis, and assess their impact on viral binding capacity and infectivity. GNE encodes the bifunctional rate-limiting enzyme for SA precursor biosynthesis [35], while SLC35A1 mediates cytidine monophosphate (CMP)-SA transport to the Golgi lumen for subsequent glycoconjugate sialylation [36]. Knockdown efficiency and its impact on SA biosynthesis were verified by qPCR and FCM. As shown in S4 Fig, gene expression analysis confirmed successful knockdown by all three crRNAs (S4A and S4B Fig), which correlated with markedly reduced surface SA levels (S4C and S4D Fig). Next, we evaluated the viral binding capacity after GNE/SLC35A1 knockdown. IF analysis demonstrated significantly attenuated viral attachment in GNE/SLC35A1 knockdown cells (Figs 4A and 4B, S4E and S4F). Quantitative analysis revealed nearly a two-fold reduction in the attachment of GCRV-I and GCRV-II particles following disruption of SA biosynthesis (Figs 4C and 4D, S4G and S4H). qPCR analysis further corroborated these findings, showing a reduction in GCRV attachment upon gene knockdown (Fig 4E-4H). To assess how SA deficiency affects post-attachment infection stages, we evaluated GCRV-I replication in GNE/SLC35A1 knockdown cells. Western blotting demonstrated significant reductions in GCRV-I protein expression (NS80, VP5, and VP7) following GNE/SLC35A1 knockdown (Fig 4I and 4J). Additionally, plaque assays demonstrated that the plaque numbers (Fig 4K and 4L) formed by the supernatants of infected cells, as well as the calculated virus titers (Fig 4M and 4N), were markedly decreased. Furthermore, viroplasms formation were significantly impaired in GNE/SLC35A1 knockdown cells (Fig 4O and 4P). Quantitative analysis from 15 infected cells in each group showed that mean number of viroplasm puncta was significantly decreased when the two genes were knockdown (Fig 4Q and 4R). Collectively, these data demonstrate that SA deficiency significantly impaired GCRV binding capacity and infectivity, establishing SA as an essential host factor throughout the viral life cycle.

### Enhanced SA biosynthesis promotes GCRV binding capacity and infectivity

**Fig 5:**
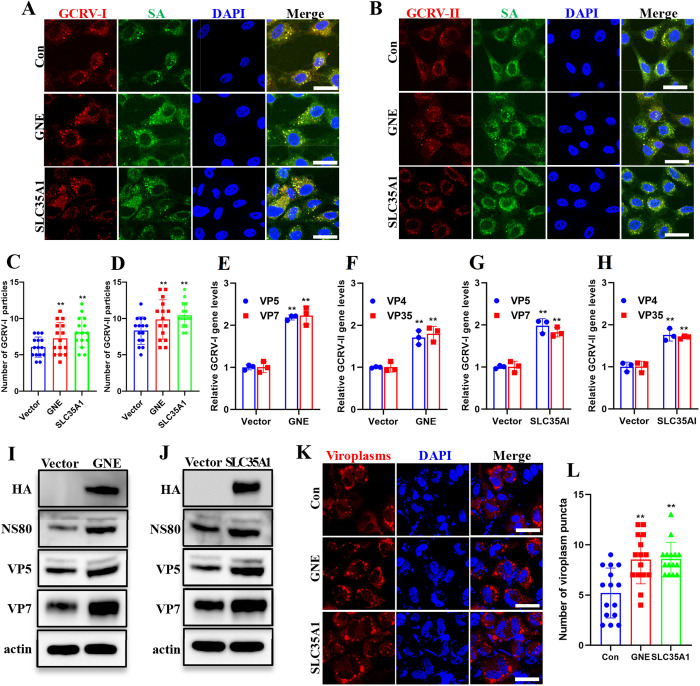
Enhanced SA biosynthesis promotes GCRV binding capacity and infectivity. **(A, B)** Immunofluorescence analysis of control or SA biosynthesis elevated cells incubated with GCRV. The control or SA biosynthesis elevated cells were incubated with GCRV-I (A) or GCRV-II (B) (MOI = 100, 1h, 4 °C), then cells were stained with FITC-conjugated WGA combined with antibodies against GCRV outer capsid proteins (VP5 for GCRV-I; VP35 for GCRV-II). Scale bar = 10 µm. **(C, D)** Quantification of attached GCRV-I (C) or GCRV-II (D) particles using ImageJ software. software. **(E-H)** RT-qPCR analysis of the relative GCRV gene levels in control or SA biosynthesis elevated cells incubated with GCRV-I (E, F) or GCRV-II (G, H). **(I, J)** Western blotting analysis of NS80, VP5, and VP7 in control or GNE- (I) or SLC35A1-overexpressed (J) cells infected GCRV-I. **(K)** Immunofluorescence analysis of the viroplasms in control or SA biosynthesis elevated cells infected with GCRV-I. Cells were infected with GCRV-I (MOI = 1, 24h, 28°C) and viroplasms were stained with antibody against NS80. Scale bar = 10 µm. **(L)** Quantitative analysis of viroplasm puncta in control or SA biosynthesis elevated cells. Data are represented as mean (n = 15 for C, D, and L, n = 3 for E-H) ± SD. ** indicates *P* < 0.01.

To complement our knockdown studies, we overexpressed GNE and SLC35A1 to enhance SA biosynthesis, then systematically assessed the impact on GCRV binding capacity and infectivity. FCM confirmed elevated surface SA levels in GNE/SLC35A1 overexpressed cells (S4I and S4J Fig). Therefore, viral binding capacity and infectivity assays were performed in GNE/SLC35A1 overexpressed cells. IF analysis demonstrated that GNE/SLC35A1 overexpression enhanced the binding capacity of both GCRV genotypes (Fig 5A and 5B). Viral particles quantification revealed GNE/SLC35A1 overexpression significantly increased attached GCRV virions (Fig 5C and 5D). Consistent with IF imaging data, qPCR revealed significantly elevated viral RNA in GNE/SLC35A1 overexpressed cells (Fig 5E-5H). Western blotting analysis revealed GNE/SLC35A1 overexpression significantly increased GCRV-I protein expression (NS80, VP5, and VP7) (Fig 5I and 5J). Furthermore, viroplasms formation were significantly enhanced in GNE/SLC35A1verexpressed cells. (Fig 5K and 5L). Collectively, these data demonstrate that enhanced SA biosynthesis promotes GCRV binding capacity and infectivity.

### The direct binding between GCRV capsid proteins and soluble SA

**Fig 6:**
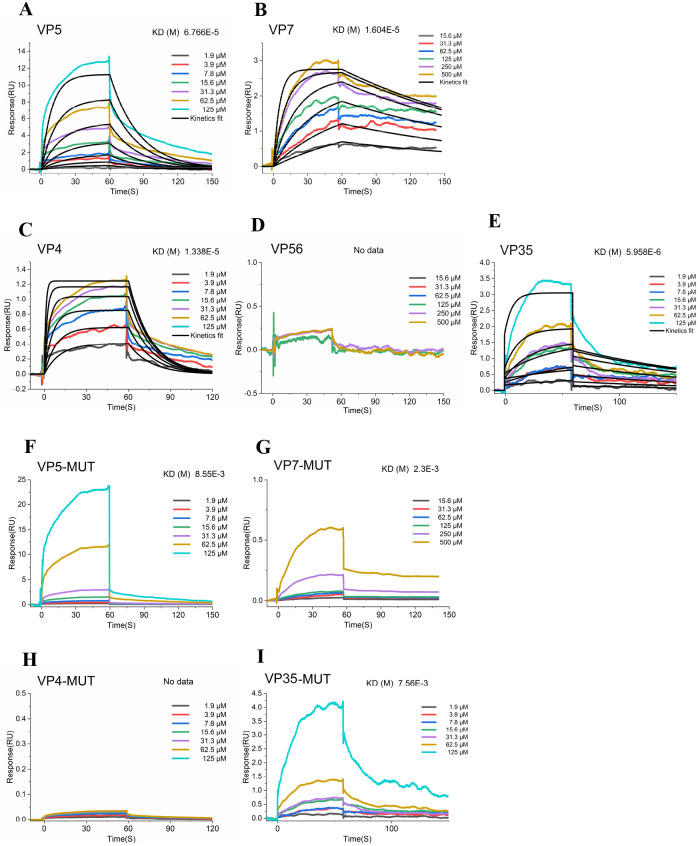
The direct binding between GCRV capsid proteins and soluble SA. **(A-E)** SPR analysis of the interactions between soluble SA and GCRV outer capsid proteins VP5 (A), VP7 (B), VP4 (C), VP56 (D), and VP35 (E). **(D-G)** SPR analysis of the interactions between soluble SA and mutant forms VP5 (F), VP7 (G), VP4 **(H)**, and VP35 **(I)**.

While our findings establish SA as a critical host factor facilitating GCRV binding capacity and infectivity, the precise molecular interactions between viral proteins and SA require further characterization. Structural and functional studies have established that reoviral outer capsid proteins mediate critical early infection steps, including receptor binding and membrane penetration [37]. To elucidate the molecular basis of SA-mediated attachment, we characterized the specific interactions between soluble SA and GCRV outer capsid proteins (VP5/VP7 for GCRV-I; VP4/VP56/VP35 for GCRV-II). We purified these capsid proteins and analyzed their interaction with soluble SA (Neu5Ac) by surface plasmon resonance (SPR) assay. As shown in Fig 6, for the two GCRV-I outer capsid proteins, both interact with soluble SA, with equilibrium dissociation constants (KD) of 6.766E-5 M and 1.604E-5 M, respectively (Fig 6A and 6B). However, for the three outer capsid proteins of GCRV-II, only VP4 and VP35 bind to soluble SA, with KD values of 1.338E-5 M and 5.958E-6 M, respectively (Fig 6C and 6E). In contrast, the outer capsid protein VP56 showed no binding signal with soluble SA (Fig 6D). In summary, these results demonstrate direct binding between GCRV capsid proteins and soluble SA, providing biophysical evidence that SA mediates viral attachment through molecular interactions.

To further explore the binding mechanisms, we utilized molecular docking analysis to predict key interaction sites on VP5, VP7, VP4, and VP35 involved in binding with soluble SA. Our analysis identified several critical residues: five in VP5 (Ile357, Ser455, Asn456, Thr458, Asp461), three in VP7 (Ala211, Cys237, Gly239), five in VP4 (Asn3, Thr6, Lys202, Gln205, Asn206), and three in VP35 (Ser153, Trp155, Glu170) crucial for SA binding (S5A-S5D Fig). We generated plasmids with mutations of these residues to alanine and purified the resulting mutant proteins. Our findings confirmed that these mutants exhibited very low affinity or no binding signal to soluble SA, underscoring the pivotal role of these residues in the interaction between GCRV capsid proteins and SA (Fig 6F-6I).

### Pretreatment of GCRV with soluble SAs increased grass carp survival rate after infection

**Fig 7:**
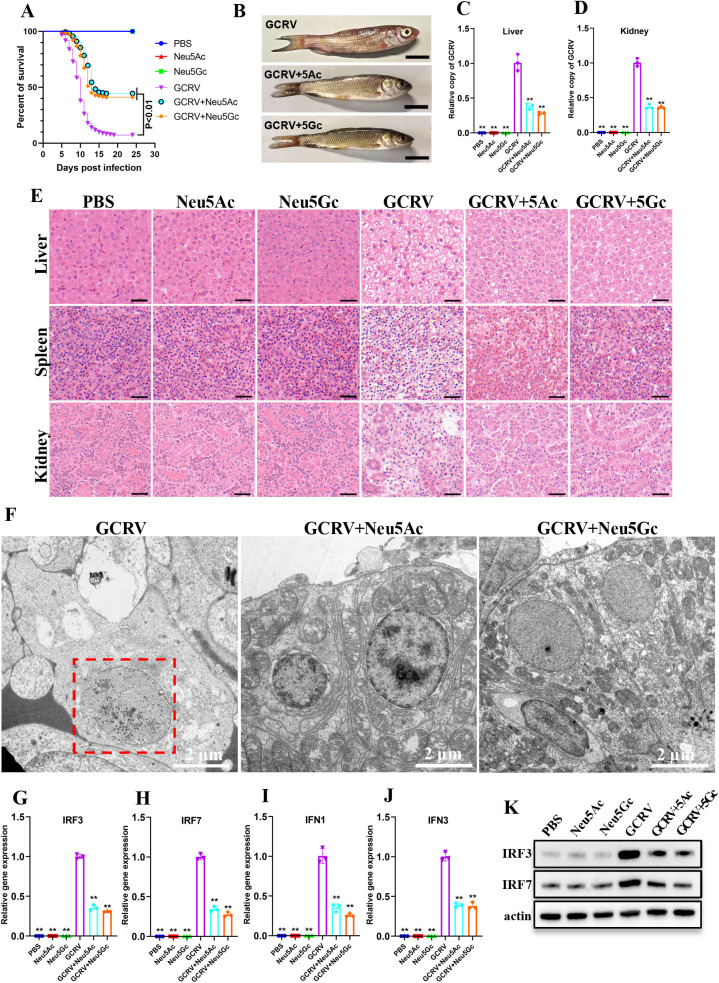
Pretreatment of GCRV with soluble SAs increased grass carp survival rate after infection. **(A)** Survival percentage of in six grass carp groups. **(B)** The hemorrhagic symptoms in fish infected with GCRV-II or with SA-preincubated GCRV-II. Scale bar = 1 cm. **(C, D)** RT-qPCR analysis of the relative GCRV-II copy number in liver (C) and kidney (D) from different grass carp groups. **(E)** Histological section analysis of the liver, spleen, and kidney samples from different groups. Scale bar = 20 µm. **(F)** TEM analysis of kidney samples from fish injected with GCRV-II, GCRV-II + Neu5Ac, and GCRV-II + Neu5Gc. The red frames indicate the GCRV virions enclosed by vesicles. Scale bar = 2 µm. **(G-J)**. RT-qPCR analysis of IRF3 (G), IRF7 (H), IFN1 (I), and IFN3 (J) in different groups. **(K)** Western blotting analysis of IRF3 and IRF7 in different groups. Data are represented as mean (n = 3) ± SD. ** indicates *P* < 0.01.

We next assessed whether the soluble SA-mediated viral neutralization observed *in vitro* could confer protection against lethal GCRV challenge in grass carp. Given that GCRV-II is the dominant circulating strain in China and is highly virulent (≥80% mortality in juvenile carp) [31], we selected this genotype for challenge studies to maximize clinical relevance. GCRV-II viral stock (2.97 × 10^3^ RNA copies/μL) was pre-incubated with 100 μg/mL Neu5Ac or Neu5Gc at 4°C overnight. Following this, six fish groups (n = 180/group) received 100 µL intramuscular injections of: (1) GCRV-II suspension, (2) GCRV-II + Neu5Ac, (3) GCRV-II + Neu5Gc, (4) Neu5Ac alone (100 µg/mL), (5) Neu5Gc alone (100 µg/mL), or (6) PBS control. Fish were monitored daily, and mortality was recorded for each group. As shown in Fig 7A, there were striking differences in mortality rates between the groups: while GCRV-II infection caused 93.9% lethality by day 20 (with initial mortality at day 4), virus pretreated with SA significantly reduced mortality (Neu5Ac: 52.2%; Neu5Gc: 61.1%) and delayed onset of death (day 6). Notably, all fish in the SA-only and PBS control groups survived. Fish receiving untreated GCRV-II exhibited characteristic hemorrhaging in oral, cephalic, and dermal tissues, whereas SA-pretreated groups showed markedly attenuated symptom severity (Fig 7B). qPCR analysis revealed significantly lower GCRV-II loads in the SA-pretreated groups across all tissues examined (Fig 7C and 7D). Histopathological analysis demonstrated severe tissue damage, including necrotic lesions, vacuolization, and karyorrhexis in GCRV-II-infected fish, while SA-pretreated groups showed much milder lesions (Fig 7E). TEM images revealed abundant vesicle-enclosed GCRV virions in GCRV-II-infected kidney tissues, but almost none in the other five groups (Figs 7F and S6). Compared to fish infected with GCRV-II alone, qPCR and western blotting showed that the SA-pretreated groups had much lower expression of key immune genes, such as IRF3, IRF7, IFN1, and IFN3 (Fig 7G-7K), indicating that SA treatment not only decreased viral infection but also mitigated the immune overactivation triggered by the virus. Collectively, these results suggest that pretreatment of GCRV with soluble SAs enhances the survival rate of grass carp after infection.

## Discussion

As obligate intracellular pathogens, viruses require host cell entry to complete their life cycle. This process begins with binding to cell surface receptors, a critical step that determines viral host range and tissue tropism. Grass carp reovirus (GCRV), the most pathogenic member of the *Aquareovirus* genus (*Spinareoviridae* family) [2], causes severe hemorrhagic disease and threatens China’s grass carp aquaculture industry. Although GCRV has been extensively studied [38], its cellular receptors remained unidentified. Our work reveals that SA acts as a key functional receptor for both GCRV-I and GCRV-II. This discovery provides a potential target for developing disease-resistant grass carp strains and new strategies to combat GCRV infection.

SAs are derivatives of neuraminic acid, a nine-carbon monosaccharide that terminates glycan chains of asparagine (N-) or serine/threonine (O-) linked glycoproteins and glycolipids, imparting a net negative charge to these structures [39,40]. Many viruses exploit SA as a receptor to mediate initial attachment to host cell surfaces [35,41,42]. SA structures exhibit remarkable diversity, with over 50 variants arising from modifications such as acetylation or methylation of hydroxyl groups [43]. In mammals, the most common forms are Neu5Ac and Neu5Gc [44]. SAs are further diversified by their linkages: they typically attach via α2,3- or α2,6-linkages to Gal or GalNAc residues, referred to as α2,3- or α2,6-linked SA, respectively (39). Additionally, SAs can also form α2,8-linkages with other SA residues, further contributing to their structural complexity. The ubiquitous expression and structural diversity of SAs on eukaryotic cell surfaces make them a key target for pathogen attachment, facilitating host-cell interactions.

To confirm whether SA serves as a functional receptor for GCRV, we investigated its role in infection through multiple experimental approaches. First, we removed cell surface SAs using two different neuraminidases, both of which significantly reduced GCRV attachment and infection. Additionally, competitive binding assays with three lectins demonstrated a marked impairment in GCRV binding to cells. Pretreatment of GCRV with two soluble SAs also effectively inhibited its ability to attach to cells and initiate infection. To further explore this, we manipulated SA biosynthesis by knocking down or overexpressing two key genes, GNE and SLC35A1, which regulate SA production. Knockdown of these genes reduced SA levels, inhibiting GCRV binding and infectivity, while their overexpression increased SA biosynthesis, facilitating viral attachment and infection. Importantly, we confirmed the direct binding between SA and GCRV capsid proteins using surface plasmon resonance (SPR). Finally, a viral challenge in grass carp demonstrated that pretreatment of GCRV with soluble SAs significantly improved survival rates in infected fish. Collectively, these findings provide strong evidence that GCRV utilizes SA as a functional receptor for host cell attachment.

Since viral envelope/capsid proteins mediate receptor binding (13,14), we characterized direct interactions between GCRV capsid proteins and soluble SA using biochemical assays. SPR assays demonstrated that both outer capsid proteins of GCRV-I directly interact with soluble SA. In contrast, among the three outer capsid proteins of GCRV-II, only VP4 and VP35 exhibited SA-binding activity. These results reveal genotype-specific differences in SA recognition, suggesting distinct attachment mechanisms between GCRV-I and GCRV-II. Mammalian reovirus (MRV) also utilizes SA as a receptor for virus attachment and entry, with the interaction mediated by the σ1 outer capsid protein [45]. However, other outer capsid proteins, such as σ3 and λ2, are also involved in the interaction between MRV and other receptors [46,47]. Interestingly, VP56, a homolog of MRV σ1 protein [48], showed no binding signal with soluble SA. These results indicate that reovirus outer capsid proteins may play diverse role in binding to different receptors. This difference in binding patterns between MRV and GCRV suggests that these viruses may utilize distinct mechanisms for attachment and entry.

**Fig 8:**
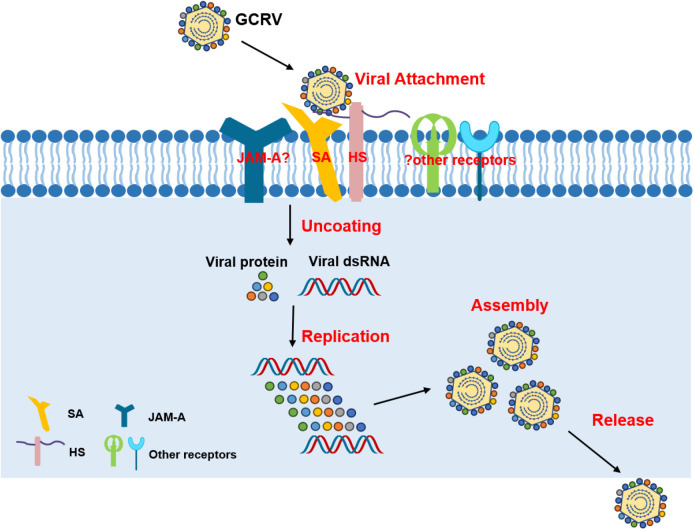
Schematic diagram of GCRV attachment and entry into host cells. The attachment and entry of GCRV may involve cooperative interactions between SA and other, as yet unidentified, receptors or co-receptors. SA: sialic acid, HS: surface heparan, JAM-A: junctional adhesion molecule A.

Although our data demonstrate that SA play a significant role in GCRV attachment and infection, several lines of evidence suggest that they are not absolutely essential for GCRV infection: (1) removal or blocking of SA reduced, but did not eliminate, viral attachment and infection; (2) treatment with different SA-binding lectins significantly, but not completely, prevented viral attachment and infection; and (3) soluble SA pretreatment increased survival rates but did not guarantee survival for all fish. These findings suggest that GCRV likely utilizes additional receptors, similar to mammalian *orthoreovirus* (MRV), which employs multiple co-receptors alongside SA [45–47,49]. Our recent study suggested that heparan sulfate (HS) also serves as an attachment receptor for GCRV [50]. Moreover, junctional adhesion molecule A (JAM-A), a well-characterized entry receptor for MRV [51], has also been reported to play an important role in GCRV infection [52,53], suggesting that it may be a candidate receptor for GCRV. Given the close phylogenetic relationship between *aquareoviruses* and *orthoreoviruses* [2,54], we propose that GCRV entry may involve cooperative interactions between SA, HS, and other unidentified receptors (Fig 8). This could explain why GCRV exhibits limited tropism despite the ubiquitous expression of SA and HS. Further studies are needed to identify these potential co-receptors and elucidate their precise roles in GCRV entry.

In conclusion, our study highlights the crucial role of cell surface SA in GCRV attachment and provides definitive evidence for SA as a functional receptor for the virus. These results present a promising target for breeding disease-resistant grass carp and developing strategies for the prevention and control of grass carp hemorrhagic disease.

## Materials and methods

### Ethics approval

All animal procedures followed ARRIVE guidelines and were approved by the Ethics Committee of the Institute of Hydrobiology, Chinese Academy of Sciences (CAS) (protocol code: IHB2023–0810). Fish underwent anesthesia with MS-222 during procedures, and we implemented strict measures to reduce discomfort.

### Cell culture and virus preparation

Grass carp ovary (GCO) and *Ctenopharyngodon idella* kidney (CIK) were maintained in M199 medium (HyClone) with 10% FBS and antibiotics (100 IU/mL penicillin, 100 μg/mL streptomycin) at 28°C with 5% CO_2_. All cell lines were routinely screened for mycoplasma contamination by PCR. For viral studies, we used two GCRV subtypes (I and II) previously isolated by our lab [31], along with *Rana grylio* virus (RGV, *Iridoviridae* family) [32,33] provided by Professor Fei Ke (Institute of Hydrobiology, CAS) as a control virus.

### Antibodies and reagents

Polyclonal antibodies against grass carp IRF3, IRF7, GCRV-I VP5, GCRV-I NS80, and GCRV-II VP35 were generated in rabbits using standard protocols. Briefly, each target gene was cloned into pET-32a, expressed in E. coli BL21 (induced with 1 mM IPTG at 20°C for 10 h), and purified via His-tag affinity chromatography. The purified proteins were emulsified with Freund’s adjuvant and used to immunize rabbits following established immunization schedules. The serums were collected after immunizing the rabbit three times. Rabbit polyclonal antibodies against RGV major capsid protein (MCP) and envelope protein 2L was kindly provided by Professor Fei Ke (Institute of Hydrobiology, CAS). Alexa Fluor 594 conjugated goat anti-rabbit IgG (Cell Signaling Technology, USA), HRP-conjugated goat anti-rabbit IgG (Biosharp, China), wheat germ agglutinin lectin (WGA) (Aladdin, China), *Sambucus nigra* lectin (SNA) (Vector, USA), *Maackia Amurensis* lectin II (MAL-II) (Vector, USA), FITC-conjugated WGA (Genetex, USA), FITC-conjugated SNA (GlycoMatrix, USA), FITC-conjugated MAL-II (GlycoMatrix, USA), neuraminidase from *Arthrobacter ureafaciens* (Nacalai tesque, Japen), neuraminidase from *Clostridium perfringens* (Aladdin, China), and DAPI (Beyotime, China) were purchased from the indicated companies.

### Virus attachment and infection assay

For attachment study, CIK cells were incubated with GCRV or RGV (MOI = 100) at 4°C for 1 h, allowing viral binding but blocking entry, followed by three washes with M199 medium before qPCR and immunofluorescence (IF) analysis. For infection assay, cells were infected with virus (MOI = 1) at 28°C for 24 h to complete the viral life cycle, then processed for western blotting, plaque assay, and IF analysis.

### Neuraminidases treatment

CIK cells were treated with neuraminidases from *C. perfringens* (α2,3-SA specific) or *A. ureafaciens* (α2,6-SA specific) at varying concentrations (0–2 U/mL) for 2 h at 28°C to selectively remove different sialic acid linkages, followed by three washes with M199 medium. For viral attachment assay, neuraminidase-treated or untreated cells were incubated with GCRV or RGV (MOI = 100, 1h, 4°C), allowing viral binding but preventing entry, then analyzed by qPCR and IF. For infection assay, neuraminidase-treated or untreated cells were challenged with virus (MOI = 1, 24h, 28°C) to assess full infection cycles via western blotting, plaque assay, and IF.

### Sialic acid binding lectins treatment

CIK cells were pretreated with lectins (WGA, SNA, MAL-II) or BSA (control) at concentrations of 0–300 μg/mL for 2 h at 28°C, followed by three washes with M199 medium. For viral attachment assay, lectin- or BSA-treated cells were incubated with GCRV or RGV (MOI = 100, 1 h, 4°C) to allow binding while blocking entry, then analyzed by qPCR and IF. For infection assay, lectin- or BSA-treated cells were challenged with virus (MOI = 1, 24 h, 28°C) and assessed via western blotting, plaque assay, and IF.

### Soluble SAs incubation assay

GCRV-I, GCRV-II, or RGV (MOI = 100) were pre-incubated with Neu5Ac or Neu5Gc (0–100 mg/mL) at 4°C overnight. For viral attachment assay, the virus-SA mixtures were then added to CIK cells (MOI = 100, 1 h, 4°C), permitting binding but blocking entry, then cell-associated virus was quantified by qPCR and IF. For infection assay, cells were infected with the virus-SA mixtures (MOI = 1, 24 h, 28°C) and assessed via western blotting, plaque assay, and IF.

### Surface plasmon resonance (SPR) assay

The binding kinetics between GCRV capsid proteins and soluble SA (Neu5Ac) were characterized using surface plasmon resonance (Biacore T200) as described previously [55]. Briefly, purified capsid proteins (10-15k RU) were immobilized on a CM5 chip via amine coupling in sodium acetate buffer (pH 5.0). Neu5Ac (serially diluted in PBS, pH 7.2-7.4) was injected at 30 μL/min (60s association/180s dissociation). Data were processed using BiaEvaluation 3.0 with double-reference subtraction, and equilibrium dissociation constants (KD) were derived from steady-state fitting.

### Plasmid construction and transfection

To study the role of SA in GCRV infection, we cloned the ORFs of GNE and SLC35A1, two key genes in SA biosynthesis, from grass carp cDNA into the pCMV-Flag vector (primers in S1 Table). All resulting plasmids were validated through DNA sequencing. For functional assay, CIK cells in 6-well plates were transfected with these constructs using Lipofectamine 3000 (Thermo Fisher Scientific, USA). At 24 h post-transfection, cells were subjected to viral attachment (MOI = 100, 4°C, 1 h) or infection (MOI = 1, 28°C, 24 h) assays as described above.

### CRISPR-Cas13d-mediated gene knockdown

To further investigate SA’s role in GCRV infection, we targeted GNE and SLC35A1 using an optimized CRISPR-Cas13d system (34). The Cas13d sequence was codon-optimized, fused with an SV40 nuclear localization signal (NLS), and cloned into pcDNA3.1. CrRNAs (three per gene; S1 Table) were designed via *cas13design* [56] and expressed from a pXR003 vector with a zebrafish U6 promoter. A CrRNA targeting luciferase served as a negative control. CIK cells were co-transfected with Cas13d and CrRNA plasmids. At 24 h post-transfection, cells were subjected to viral attachment (MOI = 100, 4°C, 1 h) or infection (MOI = 1, 28°C, 24 h) assays.

### RT-qPCR

To assess viral attachment efficiency, CIK cells were exposed to GCRV or RGV (MOI = 100) at 4°C for 1 h (permitting binding but blocking entry), washed three times with M199 medium, and harvested for RNA extraction using AG RNAex Pro Reagent (Accurate Biology, China). cDNA was synthesized with HiScript III reverse transcriptase (Vazyme, China), followed by qPCR amplification on a Bio-Rad CFX system with ChamQ SYBR Master Mix (Vazyme, China). Reactions (20 µL) contained 0.8 µL of each primer (S1 Table), 1 µL cDNA template, and were run for 40 cycles (95°C/15 s, 56°C/30 s, 72°C/30 s) after initial denaturation (95°C/10 s), with melt curve analysis. Data were normalized to β-actin using the 2^-∆∆Ct^ method [57] and presented as mean (n ≥ 3) ± standard deviation (SD) of three replicates.

### Western blotting

To evaluate the impact of SA on viral infection, CIK cells were infected with GCRV or RGV (MOI = 1) at 28°C for 24 h to allow complete viral replication. Cells were lysed in RIPA buffer (Beyotime, China) on ice for 30 min, centrifuged (12,000 × g, 10 min, 4°C), and proteins were separated by 15% SDS-PAGE before transfer to PVDF membranes (Millipore, China). Membranes were blocked with 5% nonfat milk, probed with primary antibodies (1:1000, 4°C, overnight) and HRP-conjugated secondary antibodies (1:1000, room temperature (RT), 1h), with TBST washes between steps. Protein bands were visualized using an HRP-DAB chromogenic kit (Tiangen, China).

### Plaque assay

To assess the effect of SA on viral production, CIK cells were infected with GCRV (MOI = 1, 28°C, 24 h). Supernatants containing progeny viruses were harvested and used to infect fresh CIK monolayers in 12-well plates. Cells were overlaid with 0.7% soft agar and incubated until plaques became visible (24–48 hpi). After fixation with 4% paraformaldehyde, plaques were stained with 1% crystal violet for visualization and quantification.

### Immunofluorescence microscopy

CIK cells in glass-bottom dishes were infected with GCRV under two conditions: (1) attachment assay (MOI = 100, 1 h, 4°C) or (2) infection assay (MOI = 1, 24 h, 28°C). For attachment assay, cells were fixed with 4% PFA (non-permeabilized) and dual-stained with FITC-WGA (sialic acid) plus anti-VP5 (GCRV-I) or anti-VP35 (GCRV-II) to detect surface-bound virions. For infection assay, cells were fixed, permeabilized (0.2% Triton X-100), and stained with anti-NS80 to visualize cytoplasmic viroplasms. All samples were blocked with 10% goat serum (1 h, RT), incubated with primary antibodies (2 h, RT) and fluorophore-conjugated secondaries (1 h, RT), counterstained with DAPI, and imaged on a Leica SP8 STED microscope (63 × oil). The images were further analyzed with Photoshop and Image J software to quantify the number of viral particles on cell surfaces and the viroplasm puncta in the in the cytoplasm.

### Viral challenge experiment in grass carp

Healthy grass carp (average weight: 5 g, n = 1080) from a GCRV-free population were acclimated for one week under controlled conditions (26–28°C, pH 7.5-8.5, dissolved oxygen 5–10 mg/L) and randomly divided into six experimental groups (n = 180/group). Groups received intramuscular injections of: (1) GCRV-II (2.97 × 10^3^ RNA copies/µL), (2) GCRV-II + Neu5Ac (100 µg/mL), (3) GCRV-II + Neu5Gc (100 µg/mL), (4) Neu5Ac alone (100 µg/mL), (5) Neu5Gc alone (100 µg/mL), or (6) PBS control. At 5 days post-infection (dpi), five fish per group were euthanized for multi-tissue sampling (brain, kidney, liver, spleen), while remaining fish were monitored daily until mortality stabilized (no deaths for 7 consecutive days) to calculate cumulative mortality rates.

### Hematoxylin and eosin staining

Hematoxylin and eosin staining were prepared as described previously [20]. Brieﬂy, tissue samples (brain, kidney, liver, spleen) from experimental groups were fixed in Bouin’s solution (4°C, overnight), dehydrated, and embedded in HistoResin (Leica, Germany). Sections (4 μm thickness) were cut using a Leica microtome, dried on slides (42°C, overnight), stained with hematoxylin and eosin, mounted with Permount (Fisher, USA), and imaged under phase-contrast microscopy (63 × oil immersion).

### Transmission electron microscopy

Spleen samples collected at 5 days post-infection were processed for TEM by centrifugation (2,000 × g, 5 min), followed by fixation in 2.5% glutaraldehyde (24 h, 4°C) and post-fixation in 1% osmium tetroxide (2 h, 4°C). After dehydration through an ethanol gradient, tissues were embedded in Epon-812 resin overnight, sectioned to 70 nm thickness using a Leica ultramicrotome, and double-stained with uranyl acetate and lead citrate prior to imaging on an HC-1 80.0 KV Hitachi TEM system (Hitachi, Japan).

### Cell viability assay

CIK cell viability was assessed using a CCK-8 kit under three treatment conditions: (1) neuraminidases exposure (0–2 U/mL, 2 h, 28°C), (2) lectins/BSA treatment (0–300 µg/mL, 2 h, 28°C), or (3) infection with SA-pretreated virus (1 h, 4°C). Briefly, 5 × 10^3^ cells/well (96-well plate) were incubated with 10 µL CCK-8 reagent (4 h, 28°C) after treatment, and absorbance (450 nm) was measured (Bio-Rad microplate reader). Untreated cells and medium-only wells served as positive and blank controls, respectively. Data represent mean ± SD of triplicates.

### Statistical analysis

All experiments were independently repeated ≥3 times. Data are presented as mean ± SD and analyzed using one-way ANOVA or unpaired two-tailed t-tests. Statistical significance is depicted with stars (* indicates *P* < 0.05, ** indicates *P* < 0.01, ns indicates no signiﬁcant difference.).

## Supporting information

S1 Fig The distribution of SA on the surface of grass carp cells. (**A, B**) Immunofluorescence analysis of SA distribution in CIK (A) and GCO (B) cells stained with FITC-conjugated WGA, MAL-II, and SNA. Scale bar = 10 µm.
(TIF)

S2 Fig Enzymatic removal of SA markedly diminished viral attachment and subsequent infection. (**A-C**) IF analysis of the SA in mock-treated or neuraminidase-treated cells by FITC-conjugated WGA staining. Scale bar = 10 µm. (**D-F**) Flow cytometry analysis of the relative SA fluorescence intensity in mock-treated or neuraminidase-treated cells. (**G-I**) Cell viability detection of cells treated with different concentrations of neuraminidases at 28°C for 2 hours by CCK-8 assay. (**J**) Representative blow-up images of CIK cells stained for GCRV virions and SA on cell surface. The red boxes indicate the regions of interesting (ROIs) for colocalization analysis. Scale bar = 10 µm. (**K, L**) Colocalization analysis of the relationship between SA and GCRV virions on the cell surface. PCC, Pearson colocalization coefficient. (**M-O**) Relative GCRV-II gene levels in neuraminidases-treated or untreated cells incubated with GCRV-II. NCp: neuraminidase from *C. perfringens*, NAu: neuraminidase from *A. ureafaciens*. Data are represented as mean (n = 3) ± SD. ** indicates *P* < 0.01, ns indicates no signiﬁcant difference.
(TIF)

S3 Fig The role of sialic acid-binding lectins and soluble SAs in GCRV attachment. (**A-D**) Relative GCRV-II gene levels in lectin- or BSA-treated cells incubated with GCRV-II. (**E-H**) Cell viability detection of cells treated with different concentrations of SA-binding lectins or BSA at 28°C for 2 hours by CCK-8 assay. (**I-K**) Relative GCRV-II gene levels in cells incubated soluble SA-pretreated GCRV-II. (**L-N**) Cell viability detection of cells infected with soluble SA-preincubated virus by CCK-8 assay. Data are represented as mean (n = 3) ± SD. ** indicates *P* < 0.01, ns indicates no signiﬁcant difference.
(TIF)

S4 Fig Altering SA levels in cells affects GCRV binding capacity and infectivity. (**A, B**) RT-qPCR analysis of the knockdown efficiency of GNE (A) or SLC35A1 (B) by optimized CRISPR-Cas13d RNA (CrRNA) system. (**C, D**) Flow cytometry analysis of the relative SA fluorescence intensity in control or GNE- (C) or SLC35A1-knockdown (D) cells. **(E, F)** Immunofluorescence analysis of control or SLC35A1-knockdown cells incubated with GCRV-I (E) or GCRV-II (F). The control or SLC35A1-knockdown cells were incubated with GCRV-I (E) or GCRV-II (F) (MOI = 100, 1h, 4 °C), then cells were stained with FITC-conjugated WGA and antibodies against GCRV outer capsid proteins (VP5 for GCRV-I; VP35 for GCRV-II). Scale bar = 10 µm. (**G, H**) Quantitative analysis of the number of GCRV-I (G) or GCRV-II (H) particles attached to control or SLC35A1-knockdown cells. (**I, J**) Flow cytometry analysis of the relative SA fluorescence intensity in control or GNE (I) or SLC35A1 overexpressed (J) cells. Data are represented as mean (n = 3 for A-D, I, and J, n = 15 for G and H) ± SD. ** indicates *P* < 0.01.
(TIF)

S5 Fig Determination of the key interaction sites on VP5, VP7, VP4, and VP35 involved in binding to soluble SA. (**A-D**) Molecular docking analysis showing the key residues mediating the interaction between soluble SA and VP5 (A), VP7 (B), VP4 (C), and VP35 (D).
(TIF)

S6 Fig TEM analysis of kidney samples from grass carp injected with PBS, Neu5Ac, and Neu5Gc. Scale bar = 2 µm.
(TIF)

S1 Table Primer sequences used in the study. (PDF)

S1 Movie 3D Z-section reconstruction analysis of GCRV incubated cells. (MP4)

S1 Data Source data files. (XLSX)
