# Supplementary figures and images for: Sialic acid serves as a functional receptor for grass carp reovirus
Source: PLoS Pathog. 2025 Sep 5;21(9):e1013481. doi: 10.1371/journal.ppat.1013481 (PMC12431662; doi:10.1371/journal.ppat.1013481)

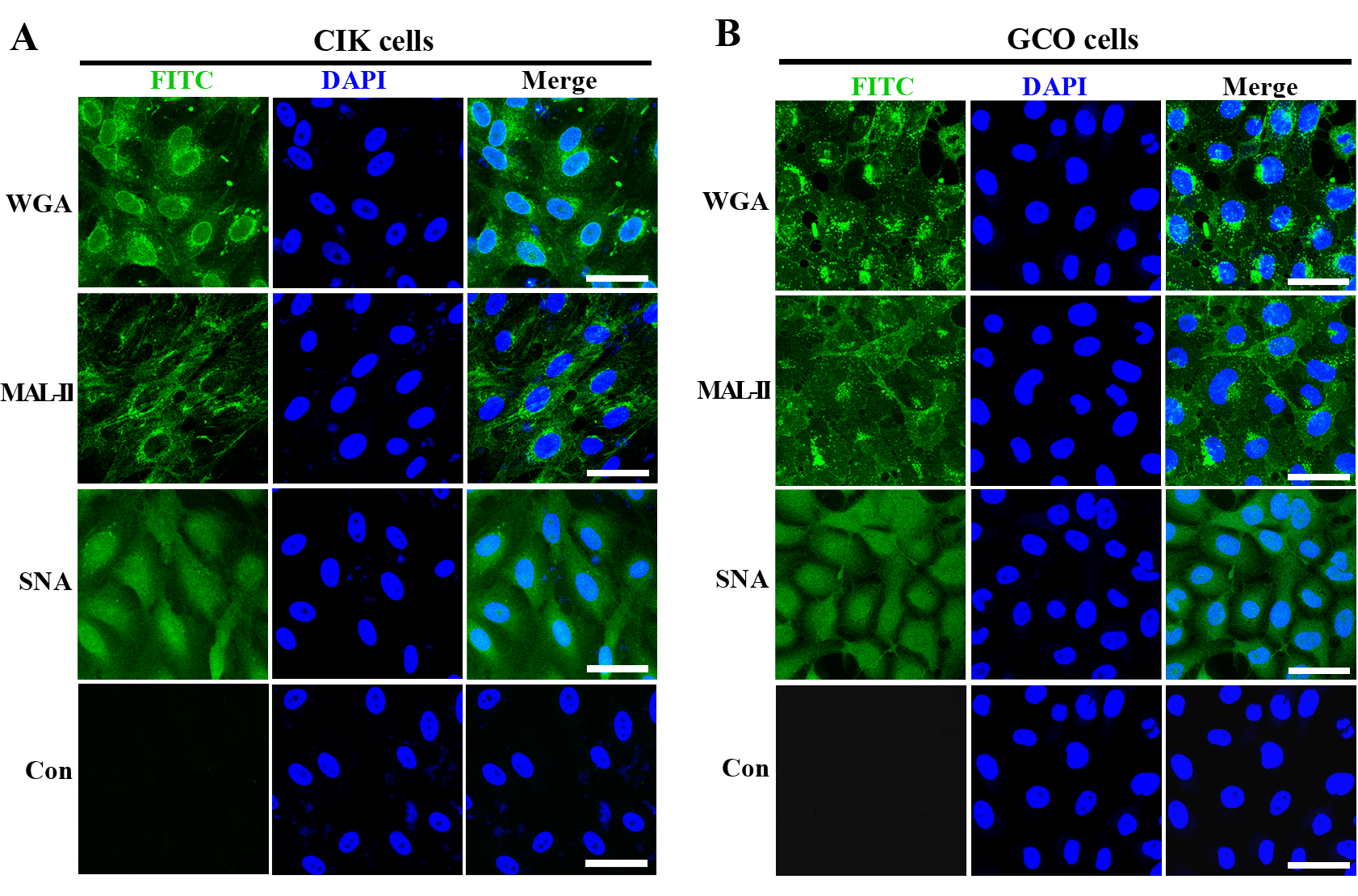

Supplement: S1 Fig — (A, B) Immunofluorescence analysis of SA distribution in CIK (A) and GCO (B) cells stained with FITC-conjugated WGA, MAL-II, and SNA. Scale bar = 10 µm. (TIF) [file ppat.1013481.s001.tif]

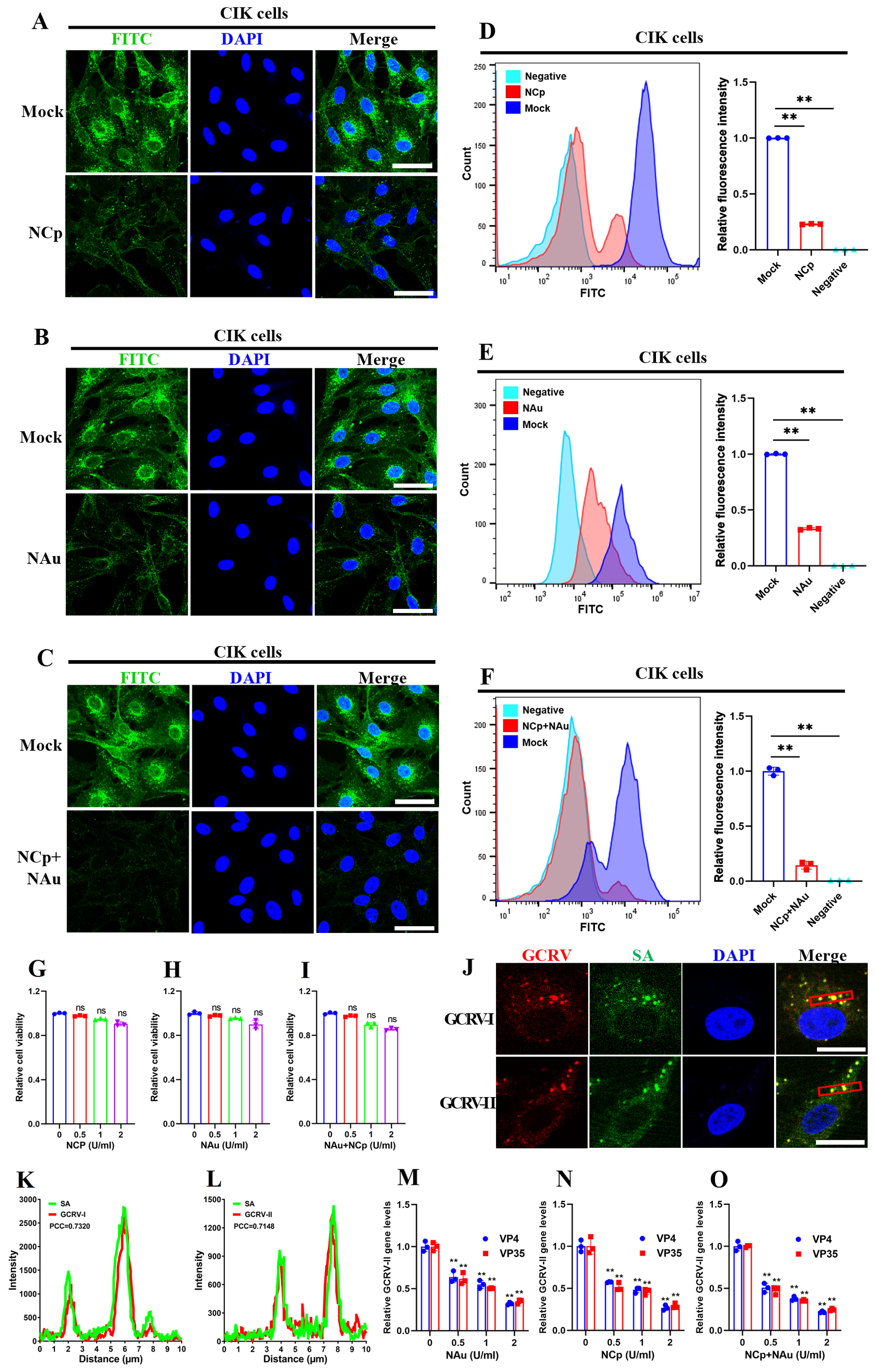

Supplement: S2 Fig — (A-C) IF analysis of the SA in mock-treated or neuraminidase-treated cells by FITC-conjugated WGA staining. Scale bar = 10 µm. (D-F) Flow cytometry analysis of the relative SA fluorescence intensity in mock-treated or neuraminidase-treated cells. (G-I) Cell viability detection of cells treated with different concentrations of neuraminidases at 28°C for 2 hours by CCK-8 assay. (J) Representative blow-up images of CIK cells stained for GCRV virions and SA on cell surface. The red boxes indicate the regions of interesting (ROIs) for colocalization analysis. Scale bar = 10 µm. (K, L) Colocalization analysis of the relationship between SA and GCRV virions on the cell surface. PCC, Pearson colocalization coefficient. (M-O) Relative GCRV-II gene levels in neuraminidases-treated or untreated cells incubated with GCRV-II. NCp: neuraminidase from C. perfringens, NAu: neuraminidase from A. ureafaciens. Data are represented as mean (n = 3) ± SD. ** indicates P < 0.01, ns indicates no signiﬁcant difference. (TIF) [file ppat.1013481.s002.tif]

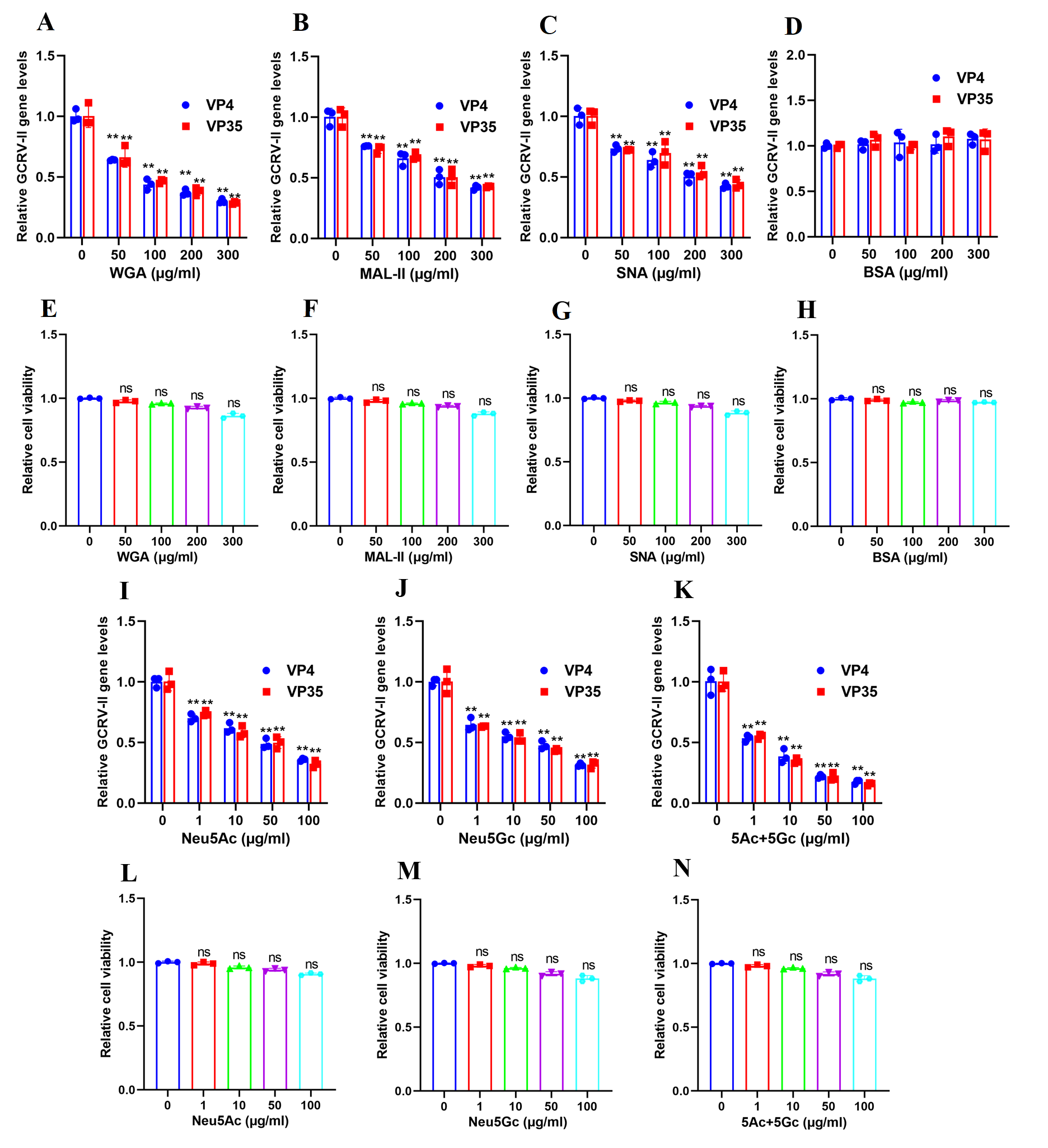

Supplement: S3 Fig — (A-D) Relative GCRV-II gene levels in lectin- or BSA-treated cells incubated with GCRV-II. (E-H) Cell viability detection of cells treated with different concentrations of SA-binding lectins or BSA at 28°C for 2 hours by CCK-8 assay. (I-K) Relative GCRV-II gene levels in cells incubated soluble SA-pretreated GCRV-II. (L-N) Cell viability detection of cells infected with soluble SA-preincubated virus by CCK-8 assay. Data are represented as mean (n = 3) ± SD. ** indicates P < 0.01, ns indicates no signiﬁcant difference. (TIF) [file ppat.1013481.s003.tif]

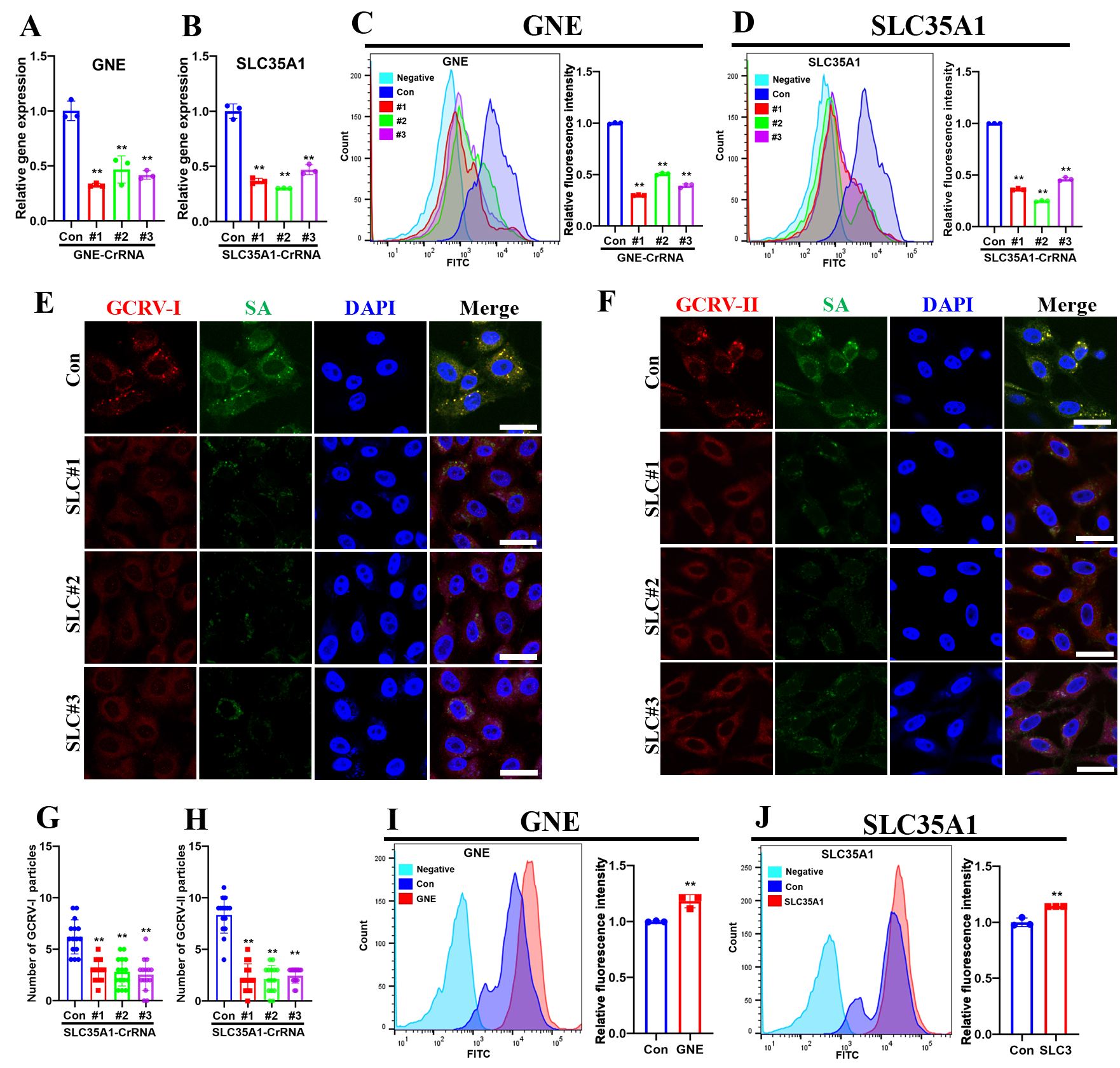

Supplement: S4 Fig — (A, B) RT-qPCR analysis of the knockdown efficiency of GNE (A) or SLC35A1 (B) by optimized CRISPR-Cas13d RNA (CrRNA) system. (C, D) Flow cytometry analysis of the relative SA fluorescence intensity in control or GNE- (C) or SLC35A1-knockdown (D) cells. (E, F) Immunofluorescence analysis of control or SLC35A1-knockdown cells incubated with GCRV-I (E) or GCRV-II (F). The control or SLC35A1-knockdown cells were incubated with GCRV-I (E) or GCRV-II (F) (MOI = 100, 1h, 4 °C), then cells were stained with FITC-conjugated WGA and antibodies against GCRV outer capsid proteins (VP5 for GCRV-I; VP35 for GCRV-II). Scale bar = 10 µm. (G, H) Quantitative analysis of the number of GCRV-I (G) or GCRV-II (H) particles attached to control or SLC35A1-knockdown cells. (I, J) Flow cytometry analysis of the relative SA fluorescence intensity in control or GNE (I) or SLC35A1 overexpressed (J) cells. Data are represented as mean (n = 3 for A-D, I, and J, n = 15 for G and H) ± SD. ** indicates P < 0.01. (TIF) [file ppat.1013481.s004.tif]

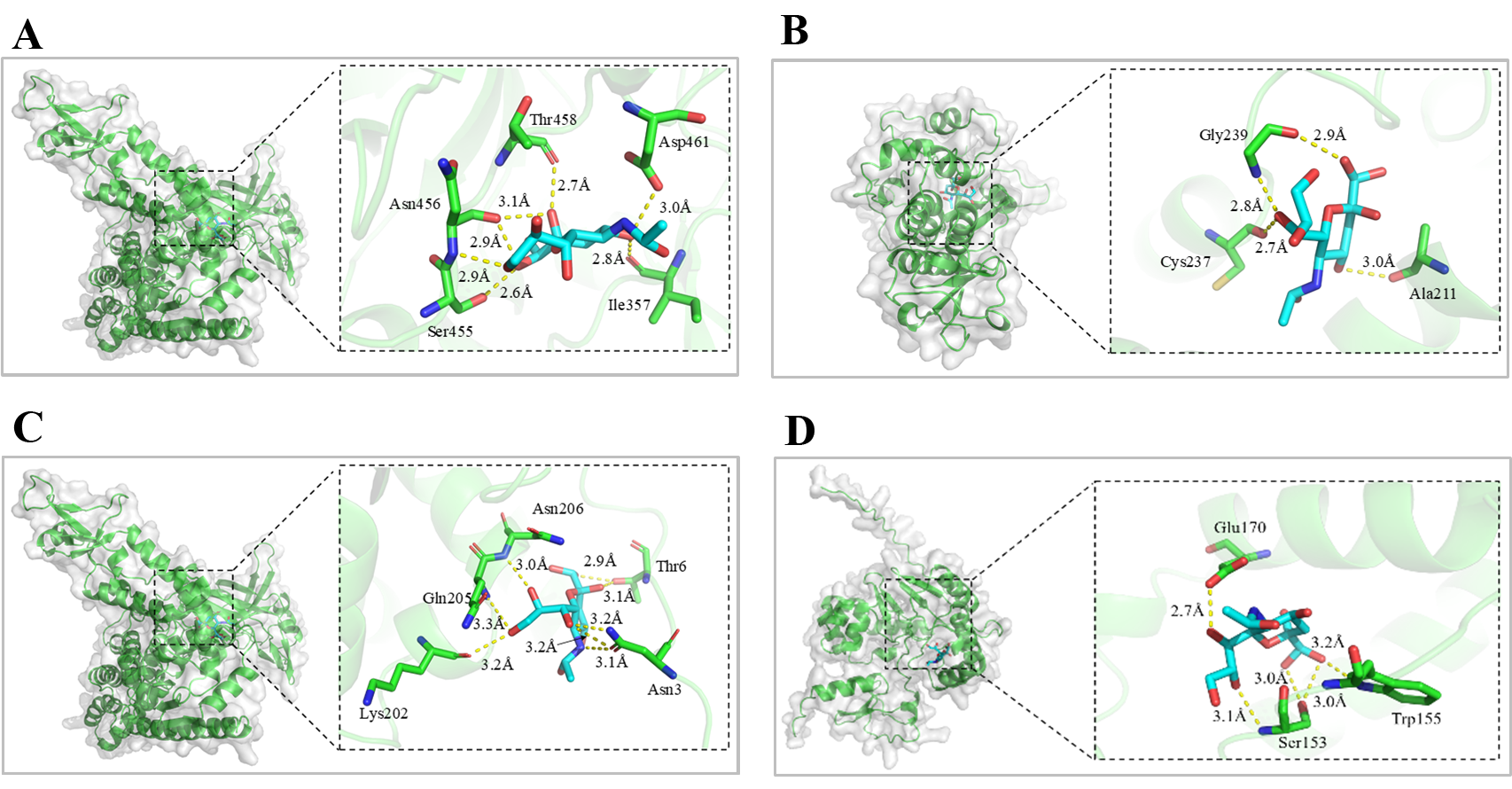

Supplement: S5 Fig — (A-D) Molecular docking analysis showing the key residues mediating the interaction between soluble SA and VP5 (A), VP7 (B), VP4 (C), and VP35 (D). (TIF) [file ppat.1013481.s005.tif]

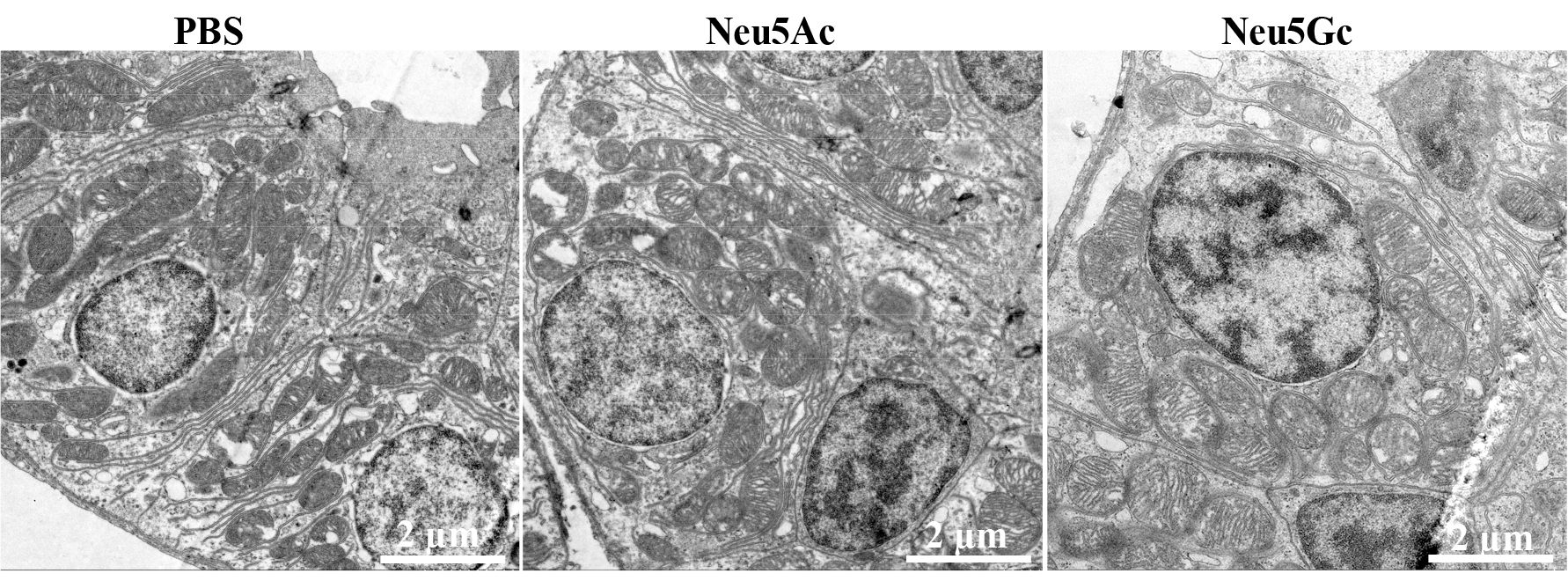

Supplement: S6 Fig — Scale bar = 2 µm. (TIF) [file ppat.1013481.s006.tif]
