# Supplementary material for: Sialic acid serves as a functional receptor for grass carp reovirus
Source: PLoS Pathog. 2025 Sep 5;21(9):e1013481. doi: 10.1371/journal.ppat.1013481 (PMC12431662; doi:10.1371/journal.ppat.1013481)
Supplement: S1 Table — (PDF) [file ppat.1013481.s007.pdf]

Table S1 Primer sequences used in the study

primers	Sequences (5' to 3')	usage
HA-GNE-F	CATGGAGGCCCGAATTCGGATGATGCCGGGACAT GCTAG	GNE Overexpression
HA-GNE-R	CGGCCGCGGTACCTCGAGTCAGTAGGTACGGCGT GTGGTATA	
HA-SLC35A1-F	CATGGAGGCCCGAATTCGGATGGCGAGCGAGTCA GTGAG	SLC35A1 Overexpression
HA-SLC35A1-R	CGGCCGCGGTACCTCGAGTCAGACGGCGATGAGT TTCTGTG	
CrRNA-Luc	AAAAGCAATTGTTCCAGGAACCA	CRISPR-Cas13d CrRNA for knockdown
CrRNA-GNE-F-#1	CTGGTCGGGGTTTGAAACTTGACATCGGCTCCAAA TTGCACTTTTTTTGAATTCTGATG	
CrRNA-GNE-F-#2	CTGGTCGGGGTTTGAAACATCTTATTGGCTGGCTG CCCGTCTTTTTTTGAATTCTGATG	
CrRNA-GNE-F-#3	CTGGTCGGGGTTTGAAACATATTGTGGCCCTGCAG CATCCTTTTTTTTGAATTCTGATG	
CrRNA-SLC35A1-F-#1	CTGGTCGGGGTTTGAAACCGGACCGTGTCCTCAGA CCTGTATTTTTTTGAATTCTGATG	
CrRNA-SLC35A1-F-#2	CTGGTCGGGGTTTGAAACAGATCCCCTGTACGGCG CTGTGCTTTTTTTGAATTCTGATG	
CrRNA-SLC35A1-F-#3	CTGGTCGGGGTTTGAAACCAGAAGTCCCAAGGAG CTGCTGATTTTTTTGAATTCTGATG	
qGNE-F	CGTTCCGCATGATTGAGCAG	QPCR of *GNE*
qGNE-R	AACGAGCAGAATGTCAGGGG	
qSLC35A1-F	CGGGTTTGCAGGTGTGTACT	QPCR of *SLC35A1*
qSLC35A1-R	CCCAAGGCGTGTATCCGTAG	
qVP4-F	AGCATCAGCAATGCAGGAGT	QPCR of *VP4*
qVP4-R	CGGGACAACATCCAAGACGA	
qVP35-F	GGTGACTGTATCCAACGGCT	QPCR of *VP35*
qVP35-R	AATGTGAGTAACCGCAGCGA	
qVP5-F	CCCGGAACAAGGCTCACCAT	QPCR of *VP5*
qVP5-R	GCGTGAGCAGTCTCCAGCTT	
qVP7-F	CGCACCTGTGACTTGGACGA	QPCR of *VP7*
qVP7-R	AGCGAGTCAGCACCTTGTCG	
qIRF3-F	AAAATGTGGACACTGACGGACC	QPCR of IRF3
qIRF3-R	CCAACACCATCTTCCCTTTGTAATA	
qIRF7-F	AATACGCTTTCCAACCACCG	QPCR of IRF7
qIRF7-R	CAGTTTTCTGGGCTCTGGGTT	
qIFN1-F	AAGCAACGAGTCTTTGAGCCT	QPCR of IFN1
qIFN1-R	CGTCCTGGAAATGACACCT	
qIFN3-F	TACATTTATAGAGACTGCGGGTGG	QPCR of IFN3
qIFN3-R	TGGAGTGTCTGGTAAACAGCCTT	
qMCP-F	GCGGTAGATCCCGTCAAGAG	QPCR of MCP
qMCP-R	AATTGGTGGATCCGGATGGG	
q2L-F	ATGTCCATCATCGGAGCGAC	QPCR of 2L
q2L-R	GCTCACCAGAGGGTCCTTAC	

qβ-actin-F	AGCCATCCTTCTTGGGTATG	QPCR of *β-actin*
qβ-actin-R	GGTGGGGCGATGATCTTGAT	
